# Potential and Challenges of *Christensenella minuta* as a Next-Generation Probiotic

**DOI:** 10.3390/foods14234085

**Published:** 2025-11-28

**Authors:** Rongrong Song, Xiaoxiao Wang, Meng Zhang, Minhao Xie

**Affiliations:** 1 College of Food Science and Engineering, Nanjing University of Finance and Economics, Nanjing 210023, China; 2 Nanjing Institute for Comprehensive Utilization of Wild Plants, China Co-ops, Nanjing 211100, China; 3 Jiangsu Province Engineering Research Center of Edible Fungus Preservation and Intensive Processing, Nanjing 210023, China

**Keywords:** *Christensenella minuta*, next-generation probiotic, gut microbiota, metabolic health, bile acid metabolism

## Abstract

*Christensenella minuta*, a Gram-negative, strictly anaerobic gut bacterium, has emerged as a promising next-generation probiotic due to its strong association with leanness and metabolic health. This review synthesizes current evidence on its multifaceted benefits, including the regulation of lipid and glucose homeostasis via bile acid modulation and short-chain fatty acid production, immunomodulation through NF-κB pathway inhibition, and the enhancement of gut-barrier integrity. Additionally, *C. minuta* demonstrates protective roles in liver injury, gut–brain-axis communication, and polycystic ovary syndrome via butyrate-mediated mechanisms. However, challenges such as oxygen sensitivity during production, strain-specific effects, and limited long-term safety data hinder clinical translation. Future research must prioritize optimized cultivation, rigorous clinical trials, and strain-level characterization to harness its full therapeutic potential for metabolic and inflammatory diseases.

## 1. Introduction

The human gut microbiota, a complex and dynamic community of microorganisms residing in the gastrointestinal tract, has emerged as a pivotal regulator of host health and disease. Its profound influence spans metabolic regulation, immune modulation, and the maintenance of gut-barrier integrity, thereby significantly impacting systemic physiological homeostasis [1,2]. Dysbiosis, or the imbalance of this microbial ecosystem, has been implicated in a wide array of disorders, including metabolic syndrome, obesity, diabetes, inflammatory bowel diseases, neurodegenerative conditions, and even cancer [3,4,5]. Given this extensive involvement, the modulation of the gut microbiota has become a promising strategy for improving health outcomes and managing various diseases.

Among the approaches to regulating the gut microbiome, the use of probiotics, live microorganisms that confer health benefits to the host when administered in adequate amounts, has gained considerable attention [6]. Traditionally, probiotics have encompassed genera such as *Lactobacillus* and *Bifidobacterium*, which are well documented for their roles in enhancing gut-barrier function, modulating immune responses, and inhibiting pathogenic bacteria colonization [7,8]. Despite their well-documented benefits, traditional probiotics involve certain limitations in stain diversity, generic effects, survivability and colonization challenges, and individual variability [2]. However, the concept and scope of probiotics have evolved significantly. Advances in microbiome research have led to the identification of novel bacterial taxa with potential probiotic properties, often referred to as next-generation probiotics (NGPs). NGPs, conforming to the normal definition of a probiotic, obviously, are typically novel or newly identified bacterial strains that are specifically isolated from the human gut microbiota and developed for targeted therapeutic purposes [9]. Unlike traditional probiotics, which are often used for general digestive wellness, NGPs are being researched and developed to prevent, treat, or cure specific diseases [10]. These include less conventional species that exhibit unique metabolic and immunomodulatory functions, and even species that are employed to improve cancer immunotherapy, offering new avenues for therapeutic interventions [2,11,12]. Many NGPs are commensal bacteria, which means that they are native, beneficial residents of a healthy human gut. They are often identified using advanced technologies, such as metagenomics and bioinformatics, by comparing the gut microbes of healthy individuals versus those with a specific disease [13]. Examples of potential NGPs include *Akkermansia muciniphila*, *Bacteroides fragilis*, *Faecalibacterium prausnitzii*, *Christensenella minuta*, *Roseburia* spp., etc. [12,14]. NGPs also fit well within the definition of live biotherapeutic products (LBPs) in that these live organisms are applicable to prevent, treat, or cure a disease or condition of human beings and are not a vaccine [9,15]. In addition, engineered live biotherapeutics are also classified as NGPs [16].

Among these emerging candidates, *C. minuta*, a Gram-negative, strictly anaerobic bacterium first identified in the human gut, has attracted substantial interest. Its relative scarcity yet consistent association with a lean host phenotype and favorable metabolic profiles distinguishes it from many other gut microbes. Notably, *C. minuta* abundance inversely correlates with obesity and metabolic syndrome, suggesting a protective role against these conditions [17]. It has been widely detected along the human gastrointestinal tract, including colonic mucosa, the ileum, the appendix, and feces, as well as possible airway colonization [18,19]. Age-related shifts in gut-microbiota composition affect *C. minuta* levels, with early life colonization influenced by maternal microbial transmission and environmental exposures, as demonstrated in animal models where maternal interventions promoted *Christensenella*-dominated enterotypes, enhancing offspring gut development [20]. Disease states, including chronic kidney disease and rheumatoid arthritis, are associated with altered *C. minuta* abundance, which could show a depletion correlating with dysbiosis and inflammation [21,22]. The unique ecological niche of *C. minuta*, coupled with its ability to interact with other gut microbes through cross-feeding and metabolic complementation, underscores its potential as a keystone species in maintaining the gut-microbial balance and host metabolic health [23]. Furthermore, recent studies have demonstrated that *C. minuta* exhibits oxygen tolerance, a trait advantageous for its cultivation and commercial probiotic application, addressing one of the major challenges in translating anaerobic gut microbes into therapeutic products [17].

The surge in interest surrounding *C. minuta* and other non-lactic acid bacteria NGPs is fueled by advancements in microbiome sequencing, culturomics, and metabolomics, which have enabled a more comprehensive understanding of their biology and host interactions [2,24,25]. These technologies facilitate the isolation, characterization, and functional assessment of such microbes, paving the way for their development as NGPs with targeted health benefits. However, despite promising preclinical and observational data, several technical and clinical challenges remain. These include optimizing cultivation methods, ensuring strain stability and viability, establishing safety profiles, understanding host-specific responses, and conducting rigorous clinical trials to substantiate efficacy [2,17].

In this context, the present review aims to systematically synthesize current knowledge on *C. minuta*, encompassing its biological characteristics, functional potential, and probiotic applications. We focus on delineating the mechanisms that underlie its beneficial effects, evaluating its role within the gut-microbial network, and highlighting hurdles and future directions concerning its clinical translation. To this end, this review is structured as follows: Section 2 details the taxonomic, genomic, and ecological features of *C. minuta*, establishing its foundational biology. Section 3 critically examines its health benefits, dissecting the mechanisms behind its effects on metabolic homeostasis, immune function, gut-barrier integrity, and systemic axes (gut–liver, gut–brain, and gut–ovary). Finally, Section 4 discusses the challenges, risks, and future perspectives for translating *C. minuta* into a safe and effective biotherapeutic. By addressing these aspects, we seek to provide a comprehensive framework that supports the advancement of *C. minuta* as a promising NGP, contributing to the broader goal of microbiota-targeted therapies for metabolic and inflammatory diseases.

## 2. Biology and Ecological Niche of *C. minuta*

### 2.1. Taxonomic and Genomic Features

**Figure 1:**
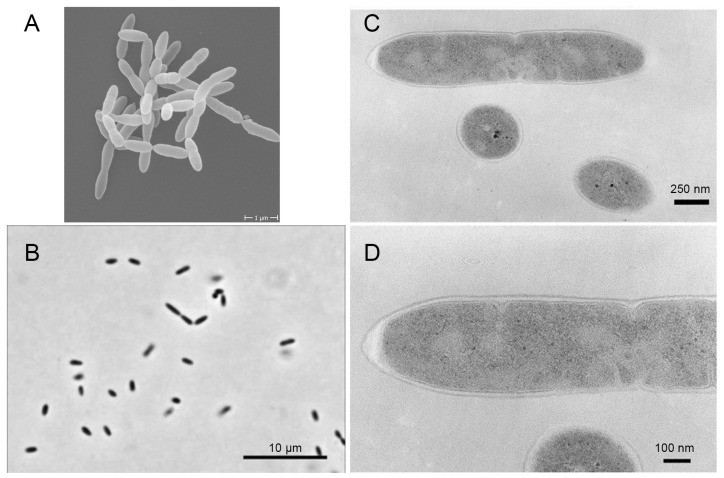
Cell morphology of *Christensenella minuta*. (**A**) Electron microscopy image; (**B**) phase-contrast micrograph; (**C**) TEM image of ultrathin-sectioned cells; (**D**) enlargement of (C) showing the cell-wall structure [18,26].

*Christensenella minuta* was first isolated from human feces and reported in 2012 [26]. It belongs to the phylum Firmicutes, class Clostridia, and order Clostridiales, and it is the type species of the proposed family Christensenellaceae. As shown in Figure 1, *C. minuta* morphologically exhibits short, straight rods with tapered ends, typically measuring 0.4 μm in width and 0.8–1.9 μm in length, occurring singly or in pairs. The transmission electron micrograph of ultrathin sections reveals details of the cell-wall structure, consistent with its Gram-negative classification [26]. Taxonomically, this microorganism is characterized by strictly anaerobic, non-motile, and non-spore-forming cells and a Gram-negative cell-wall structure [26]. Its cell-wall composition includes specific amino acids, such as glutamic acid, serine, alanine, and LL-diaminopimelic acid, alongside whole-cell sugars comprising ribose, rhamnose, galactose, and glucose. Dominant fatty acids include iso-C15:0, C16:0, and C14:0, while respiratory quinones are absent, underscoring its anaerobic metabolic adaptations [26]. Biochemically, it is negative for catalase, oxidase, urease, aesculin hydrolysis, gelatin hydrolysis, indole production, and nitrate reduction and is positive for acid production from glucose, L-arabinose, L-rhamnose, D-xylose, and salicin [26,27]. *C. minuta* can also metabolize N-acetyl-D-glucosamine, D-arabitol, arbutin, D-cellobiose, dextrin, D-fructose, L-fucose, D-galactose, maltotriose, D-mannitol, D-mannose, 3-methyl-D-glucose, palatinose, turanose, fumaric acid, pyruvic acid, L-phenylalanine, 2′-deoxyadenosine, inosine, and uridine [28]. However, the microbiological characterization of *C. minuta* could be strain-dependent. For example, strain DSM 33715 is negative for salicin [29]. Phylogenetically, based on 16S rRNA gene sequence, *C. minuta* occupies an isolated evolutionary position, forming a deep branch within Clostridiales and showing the closest affinity to *Caldicoprobacter oshimai* with 86.9% sequence similarity. Genomic analyses of multiple strains reveal conserved features. The *C. minuta* type strain DSM 22607 (=YIT 12065 = JCM 16072) comprises one circular chromosome of 2.97 Mbp and exhibits a G+C content of 51.4 mol% [26,30], while strains CIP 112228 and CIP 112229 isolated from healthy humans were fully sequenced, consisting of one circular chromosome (2.77 Mbp, 51.87 mol% GC) and confirming typical genome architecture for the species [31]. Genomic annotation revealed a significant expansion of genes in the DSM 22607 strain that are involved in carbohydrate metabolism, particularly multiple homologs of the ribose ABC transport system components, such as *RbsA*, *RbsB*, and *RbsC* [32]. This expansion may facilitate nutrient acquisition and potentially support quorum-sensing mechanisms within the gut environment [32]. Functional annotations identify a glycine-specific bile salt hydrolase (BSH; *bshA* gene) in *C. minuta* DSM 33407, preferentially deconjugating glycine-conjugated bile acids, such as glycocholic acid. A phylogenetic analysis indicated that this BSH shares less than 70% amino acid identity with other known BSHs from human gut microbiota, forming a distinct evolutionary clade [33]. Genes associated with lipopolysaccharide (LPS) biosynthesis, predicted genes *lpxA*, *lpxD*, and *lpxH*, were also identified in the genome of *C. minuta* DSM 22607 [30]. However, the LPS structure of *C. minuta*, an atypical banding pattern with reduced O-antigen content, which is correlated with genomic differences in key biosynthesis genes, differs from that of typical pathogens, including *Escherichia coli*, *Salmonella enterica*, and *Pseudomonas aeruginosa*. It exhibits weak immunostimulatory activity and induces only a mild inflammatory response in macrophages, requiring concentrations 10 to 100 times higher than *E. coli* LPS to trigger measurable NF-κB pathway activation and the production of pro-inflammatory cytokines, which will be discussed in Section 3.2 [30]. *C. minuta* exhibits significant carbohydrate utilization capabilities, producing acetate and butyrate as key metabolites, but it has a narrower carbon source utilization spectrum than most of its competitors, including *Bacteroides* and *Bifidobacterium* [23]. *C. minuta* is also capable of degrading insect chitin, enhancing short-chain fatty acid production [34]. Metagenomic studies highlight its heritable colonization correlating with metabolic health [19]. In addition, a novel D-tagatose 3-epimerase (DTEase) capable of catalyzing D-fructose into D-allulose was identified and cloned in the genome of *C. minuta* DSM 22607, as a promising candidate for industrial bioproduction [35].

### 2.2. Microbial Interactions

The abundance of *Christensenella minuta* in the human gut microbiome is highly heritable (>80% heritability) and genetically influenced by host loci, exhibiting high host-specific heritability [19]. The family Christensenellaceae is widespread across human populations, and it acts as the central hub in a co-occurrence network of other highly heritable microbes [18]. The network includes methanogenic Archaea, such as Methanobacteriaceae, and other bacterial families, and the heritable module is anti-correlated with a module containing Bacteroidaceae and Bifidobacteriaceae [19]. *C. minuta* functions as a keystone species in the gut ecosystem. Xue et al. systematically studied the interactions of *C. minuta* with multiple gut bacteria and found that *C. minuta* SJ-2 intervention reduced the diversity of fecal microorganisms significantly and specifically promoted some bacterial groups, such as Lactobacillaceae [23]. *C. minuta* exhibits metabolic auxotrophies for vitamin B1, B12, serine, and glutamate, requiring compensatory support from vitamin-synthesizing taxa to maintain ecological functionality [23,36]. *C. minuta* engages in cross-feeding symbiosis with commensal bacteria such as *Faecalibacterium prausnitzii* by supplying acetate, amino acids, and fumarate, which in turn enhance butyrate production [23]. Butyrate serves as the primary energy source for colonocytes and regulates tight junction integrity to strengthen the gut barrier, inhibits histone deacetylase, modulates gene expression, reduces pro-inflammatory cytokines, and maintains immune homeostasis [37]. *C. minuta* establishes syntrophic interactions with hydrogenotrophic archaea in the human gut, particularly *Methanobrevibacter smithii*, and their co-occurrence is driven by physical association and interspecies hydrogen transfer. *M. smithii* consumes H_2_ produced by *C. minuta* to generate CH_4_, and this metabolic cross-feeding is enhanced via the formation of dense flocs in which the archaea embed within bacterial clusters, optimizing energy harvest in anaerobic niches [38].

Conversely, *C. minuta* negatively regulates several species of *Bifidobacterium* and *Bacteroidetes*, such as *B. caccae*, *B. uniformis*, *B. fragilis*, *B. stercoris*, *B. longum*, and *B. bifidum*, due to nutrient competition. There are multiple overlaps in the majority of carbon source utilization between *C. minuta* SJ-2 and these species, as revealed by nutrient source assimilation tests [23]. Some opportunistic pathogens were specifically reduced by *C. minuta* in the co-occurrence network, such as *Klebsiella pneumoniae*, *Clostridium innocuum*, and *Streptococcus pasteurianus* [23]. *C. innocuum* showed vulnerability in the competition with *C. minuta* for lipid metabolism, as *C. innocuum* survives better in lipid-rich environments [23,39], and *S. pasteurianus* may be inhibited by the efficient SCFAs production of *C. minuta* [28]. In addition, *C. minuta* encodes BSH, catalyzing the deconjugation of primary bile acids [33]. The unconjugated bile acids exhibit significant toxicity against a broad spectrum of microorganisms, acting as key regulators of the gut-microbial community [40]. Furthermore, unconjugated bile acids can form complexes with bacterial LPS, potentially neutralizing their pro-inflammatory effects [41]. By modulating BSH activity, *C. minuta* could effectively influence the composition of the intestinal ecosystem, competitively exclude pathobionts, and contribute to maintaining intestinal homeostasis by suppressing harmful microorganisms.

### 2.3. Environmental and Dietary Modulation

*C. minuta* colonization is strongly influenced by host genetics, and its abundance is dynamically modulated by dietary interventions and environmental cues [19]. High-fat diets reduce its colonization, with obesogenic conditions diminishing *C. minuta* levels in murine models [27]. Ketogenic diet treatment increased the abundance of *C. minuta* in children with drug-resistant epilepsy, and its level correlated with seizure reduction and serum plasmalogens concentrations [42]. Compared to a soybean diet, insect-based diet (*Hermetia illucens* meal) significantly enriched *C. minuta* populations by 5-fold in laying hens, and it cooperated with *Alkaliphilus transvaalensis* and *Flavonifractor plautii*, encoding β-N-acetylhexosaminidase and N-acetylglucosamine 6-phosphate deacetylase for chitin degradation and efficiently increasing SCFAs production [34]. Environmental factors, such as bile acids and oxygen levels, have an impact on *C. minuta*. *C. minuta* expresses a glycine-specific BSH that deconjugates primary bile acids, such as taurocholic acid. It helps *C. minuta* resist bile acids (BAs), facilitating the colonization of the gastrointestinal tract environment and enabling adaptation to intestinal bile fluctuations [33,43]. While strict anaerobes, *C. minuta* could survive transient oxygen exposure, facilitating resilience in dysbiotic guts with compromised mucosal hypoxia [28]. Interventional strategies, including galacto-oligosaccharides, Procyanidin C1, alpha-tocopheryl quinone, leonurine, sea bass (*Lateolabrax maculatus*), and Huang-Qi-Ling-Hua-San, a specially designed Chinese medicine formula to treat type 2 diabetes, have been reported to elevate *C. minuta* in various animal models [20,44,45,46,47,48]. In addition, low-dose irradiation of the gut significantly increases *C. minuta* levels in patients with metastatic cancer and improves the efficacy of PD-L1 blockade [49].

## 3. Health Benefits of *C. minuta* and the Mechanisms

### 3.1. Regulating Effects on Lipid and Glucose Homeostasis

The gut bacterial family Christensenellaceae and genus *Christensenella* are consistently associated with metabolic health, but their roles and the mechanisms in benefiting host health are not fully understood. *C. minuta*, a cultured and representative member of the family, significantly modulates host lipid and glucose metabolism, and some recent studies are summarized in Table 1.

**Figure 2:**
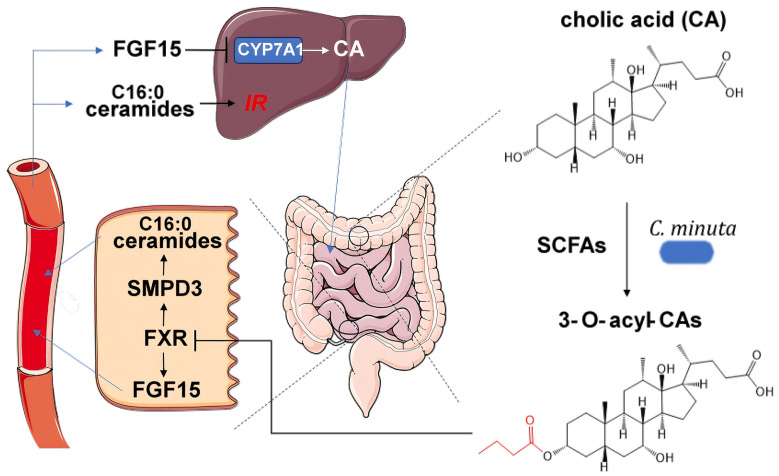
Proposed mechanism of *C. minuta* ameliorating metabolic disease by producing acylated secondary bile acids.

Amending an obesity-associated human-donor microbiome with live *C. minuta* before transplanting it into germ-free mice resulted in significantly reduced weight gain and lower adiposity in recipient mice compared to mice receiving unamended stool and altered the community structure in the mouse gut, enriching for other lean-associated bacteria like *Oscillospira* [19]. The novel strain *C. minuta* DSM33407 demonstrated significant potential as a biotherapy for obesity and associated metabolic diseases by modulating host metabolism and gut-microbial ecology [27]. In a diet-induced obesity (DIO) mouse model, daily administration of this strain prevented body weight gain, reduced adiposity, improved glycemic control, and lowered plasma levels of leptin and resistin without affecting food intake. It profoundly influenced hepatic lipid metabolism by inhibiting de novo lipogenesis, notably repressing glucokinase gene expression, which resulted in reduced hepatic triglyceride and free fatty acid accumulation [27]. Additionally, *C. minuta* DSM33407 functioned as a keystone species in the gut, shifting microbial community structure by decreasing the Firmicutes/Bacteroidetes ratio and increasing overall diversity, as evidenced in both DIO mice and a humanized simulator of the human intestinal microbial ecosystem (SHIME) model inoculated with obese microbiota. These microbial changes were associated with an enhanced production of beneficial short-chain fatty acids (SCFAs), reduced branched-chain fatty acids, and promoted the deconjugation of primary BAs [27]. Furthermore, the strain strengthened gut epithelial integrity, upregulating tight junction proteins in vivo and enhancing transepithelial electrical resistance in vitro, suggesting a protective role against metabolic endotoxemia and inflammation [27]. In another study, *C. minuta* demonstrates significant effects as a keystone gut species by markedly altering host energy homeostasis and gut-microbial ecology [50]. Amendment with live *C. minuta* DSM 22607 (compared to a heat-killed control) in germ-free mice colonized with obese-human-donor microbiota resulted in reduced feed efficiency, indicating that less weight was gained per calorie consumed, accompanied by a significant increase in voluntary physical activity in a sex-dependent manner [50]. Live *C. minuta* increased intestinal microbial biomass, and reduced within- and between-host microbial diversity, immobilizing dietary carbon within the microbiome and leading to a greater loss of energy in the stool. The microbiota remodeling was linked to metabolic changes, including altered cecal SCFAs, such as reduced butyrate in males, and shifts in host-serum metabolome pathways related to steroid hormone and BA biosynthesis in females [50]. The administration of these live *C. minuta* DSM 22607 to type-2-diabetic (T2D) mice significantly improved glycemic control and lipid metabolism, which manifested in reduced fasting blood glucose, enhanced oral glucose tolerance, and improved serum lipid profiles [47]. *Christensenella* supplementation promoted the secretion of glucagon-like peptide-1 (GLP-1), a key incretin hormone that stimulates insulin secretion. It inhibited hepatic gluconeogenesis by downregulating the expression of rate-limiting enzymes glucose-6-phosphatase (G6PC) and phosphoenolpyruvate carboxykinase (PEPCK). Crucially, it reduced intestinal glucose absorption by suppressing the expression of transporters SGLT1 and GLUT2 in the ileum. Furthermore, *Christensenella* enhanced intestinal barrier integrity, evidenced by the upregulated expression of tight junction proteins ZO-1 and Claudin-1, which consequently reduced systemic lipopolysaccharide (LPS) levels and dampened the LPS/TLR4/NF-κB inflammatory pathway. The treatment also boosted the host’s antioxidant capacity and modulated their liver metabolism, particularly by reducing the levels of detrimental branched-chain amino acids (BCAAs), tryptophan, and tyrosine. The study also positioned *C. minuta* as a critical probiotic target and a primary effector of the herbal medicine’s therapeutic action against T2D [47]. Notably, in a groundbreaking study, *C. minuta* was revealed to ameliorate metabolic disease by producing a novel class of acylated secondary BAs, specifically 3-O-acyl-cholic acids (3-O-acyl-CAs) [51]. These derivatives functioned as potent and selective antagonists of the intestinal farnesoid X receptor (FXR). The bacterium enzymatically catalyzed the acylation of the host’s primary BA, cholic acid, at the 3-hydroxyl position using short-chain fatty acyl-CoAs as substrates, generating derivatives like 3-acetylcholic acid (Ac-CA), 3-propionylcholic acid (Prp-CA), 3-butyrylcholic acid (Buty-CA), and 3-valerylcholic acid (Val-CA) [51]. The administration of live *C. minuta* to DIO mice significantly increased the levels of these 3-O-acyl-CAs in the ileum, which in turn specifically inhibited intestinal FXR signaling. This targeted antagonism disrupted two key FXR-mediated axes. It suppressed the FXR-FGF15 pathway, leading to increased hepatic *Cyp7a1* expression and BA synthesis from cholesterol, and it inhibited the FXR-Smpd3 pathway, resulting in reduced production of ceramides, particularly the C16:0 ceramide, a key driver of insulin resistance and hepatic gluconeogenesis [51]. Consequently, this multifaceted modulation resulted in markedly improved glycemic control, reduced hyperlipidemia, alleviated hepatic steatosis, and decreased inflammation. Crucially, the entire beneficial effect was abrogated in intestinal-specific FXR knockout mice, proving that the gut-specific FXR was the primary therapeutic target [51]. The multifaceted modulation, summarized and illustrated in Figure 2, resulted in markedly improved glycemic control and reduced hepatic steatosis [51]. The profound physiological relevance of this mechanism was underscored by the finding that these beneficial 3-O-acyl-CAs were significantly depleted in the feces of human patients with type 2 diabetes compared to healthy individuals [51].

**Table 1:** Effects of *C. minuta* on microbiota and metabolic disorders.

Strain	Model	Dose	Duration	Change in Microbiota	Outcome	Ref.
DSM 33407	Diet (D12451i) induced obesity mouse	2 × 10^9^ CFU/day	4–12 weeks	↓ Firmicutes/Bacteroidetes ratio	↓ body weight gain, hyperglycemia, fat mass, circulating leptin and resistin levels, hepatic lipids, and *Gck* expression levels	[27]
CGMCC 1.32672	Diet (D12492i)-induced obese mice	5 × 10^8^ CFU/day	8 weeks	↔ Alpha diversity and beta diversity ↑ Christensenellaceae and a limited number of ASVs	↓ pathoglycaemia and lipometabolic disorders, intrahepatic and colonic inflammation, fatty liver-like lesions, fat deposition and hepatocyte ballooning ↑ intestinal epithelia integrity	[51]
DSM 22607	germ-free mice with an obese human donor microbiota	1 × 10^10^ CFU/mouse	4 weeks, *C. minuta* only amended at the beginning	↑ Gut-microbial biomass ↓ Microbial diversity	↓ feed efficiency ↑ energy expenditure, physical activity	[50]
DSM 22607	high-fat diet and injected STZ induced T2D mice	3 × 10^8^ CFU/day	8 weeks	↑ *Christensenella*, *Bifidobacterium*, *Phascolarctobacterium*, *Collisella* ↓ *Muribaculum*, *Ruminiclostridium_5*, *Lachnospiraceae_FCS020_group*	↑ blood glucose and lipid metabolism, GLP-1 secretion, antioxidant capacity, intestinal barrier ↓ hepatic gluconeogenesis, intestinal glucose absorption, LPS-induced inflammation, branched amino acids content in liver	[47]

Legend: ↑, increased/enriched; ↓, decreased/reduced; ↔, no significant change. CFU, colony-forming unit; STZ, Streptozotocin; T2D, type 2 diabetes; ASV, Amplicon Sequence Variant; Gck, Glucokinase; GLP-1, Glucagon-like peptide-1.

Similar metabolic dysfunctions alleviating effects were also observed in the research about *C. tenuis*, a close relative of *C. minuta* [41]. It has been demonstrated to alleviate endotoxemia and metabolic disorders in DIO mice, by inhibiting intestinal LPS translocation and modulating related metabolic pathways. The mechanism involved the suppression of the LPS-TLR4 signaling pathway, which reduced inflammatory responses and improved metabolic outcomes [41]. Through omics analysis, it was found that treatment with *C. tenuis* increases levels of free BAs in the gut, and in vitro experiments confirmed that this bacterium hydrolyzes conjugated BAs into free BAs via BSH activity. Molecular dynamics simulations revealed that free BAs form non-membrane-permeable complexes with LPS, effectively preventing their transmembrane translocation from the intestine [41]. Experimental validation using isothermal titration calorimetry showed that free BAs bind directly to LPS in an enthalpy-driven manner, corroborating the simulation results and highlighting specific molecular interactions [41]. Additionally, the oral administration of free BAs in DIO mice resulted in reduced plasma LPS levels, further supporting the role of this pathway in mitigating endotoxemia. These findings uncovered a novel mechanism through which BSH-positive gut microbes like *C. tenuis* benefit host metabolism through BA-mediated inhibition of LPS translocation, providing a foundation for developing gut-targeted biotherapies for metabolic diseases and endotoxemia [41].

### 3.2. Modulations on Immune and Gut Barrier

*Christensenella minuta* demonstrates potent immunomodulatory properties, and some recent studies are summarized in Table 2. *C. minuta* DSM 22607 demonstrated significant anti-inflammatory and immunomodulatory properties both in vitro and in vivo, highlighting its potential as a therapeutic agent for inflammatory bowel diseases (IBDs) [28]. It reduced the TNF-α-induced secretion of the pro-inflammatory cytokine IL-8 by approximately 50% in human HT-29 cells, an effect achieved through the inhibition of the NF-κB signaling pathway in vitro, particularly via soluble effector molecules present in its supernatant [28]. Additionally, it helped maintain intestinal epithelial barrier integrity by stabilizing transepithelial electrical resistance (TEER) in Caco-2 cells exposed to TNF-α [28]. In both dinitrobenzene sulfonic acid (DNBS)-induced murine and 2,4,6-trinitrobenzenesulfonic acid (TNBS)-induced rat colitis models in vivo, *C. minuta* administration reduced macroscopic and microscopic colon damage, decreased neutrophil infiltration, as indicated by lowered myeloperoxidase activity and lipocalin-2 levels, and downregulated pro-inflammatory cytokines such as IL-1β [28]. The mechanisms were attributed to high acetate and moderate butyrate production, oxygen tolerance that enhances survivability in inflammatory environments, and the secretion of yet-unidentified anti-inflammatory compounds [28]. In a DNBS-induced colitis mouse model, *C. minuta* DSM 22607 also demonstrated significant protective and restorative effects on the intestinal barrier [52]. The bacterium markedly reduced intestinal permeability by specifically improving paracellular pathways, evidenced by a decreased flux of TRITC-dextran and FSA in the colon, indicating enhanced mucosal integrity. It counteracted goblet-cell depletion and increased mucin 2 (MUC2) expression, thereby preserving mucus-layer thickness [52]. *C. minuta* DSM 22607 effectively alleviated dextran sulfate sodium (DSS)-induced ulcerative colitis in mice by promoting mucosal healing [53]. It significantly remodeled the gut microbiota, increasing beneficial bacteria like *Dubosiella* while reducing pathogenic genera, and elevated SCFAs production, particularly the production of propionic acid [53]. These SCFAs serve as key signaling molecules, stimulating the expression of Insulin-like Growth Factor-1 (IGF-1) both systemically and locally in the colon. The increased IGF-1 levels subsequently activated PI3K-AKT signaling pathway, a central driver of cell proliferation and survival [53]. This activation fueled the regeneration and migration of intestinal epithelial cells (IECs), accelerating mucosal repair, and the effects were confirmed via the abolished therapeutic benefits upon PI3K-AKT pathway inhibition [53]. Concurrently, *C. minuta* exerted potent immunomodulatory effects by promoting the polarization of macrophages towards the anti-inflammatory, wound-healing M2 phenotype, leading to a favorable cytokine shift characterized by reduced pro-inflammatory factors (IL-6, TNF-α) and increased anti-inflammatory IL-10 [53]. In summary, *C. minuta* ameliorated colitis through an integrated mechanism that linked gut-microbiota modulation and SCFA production to the activation of the IGF-1/PI3K-AKT pathway in epithelial cells while simultaneously resolving inflammation via macrophage reprogramming [53]. These positioned *C. minuta* DSM 22607as a promising candidate for developing next-generation microbiome-based biotherapeutics aimed at restoring gut homeostasis and alleviating IBD symptoms [28,52,53].

In a comprehensive screening process to identify a potent *C. minuta* strain for Crohn’s disease therapy, *C. minuta* DSM 33715 was selected as a superior clinical candidate based on its robust anti-inflammatory and barrier-strengthening properties [29]. The strain demonstrated significant efficacy in vitro by enhancing intestinal barrier integrity, evidenced by increased transepithelial electrical resistance (TEER) in human Caco-2 cells, and reducing pro-inflammatory IL-8 production in TNF-α-stimulated HT-29 cells, while stimulating anti-inflammatory IL-10 in PBMCs and macrophages [29]. Validation in vivo in TNBS-induced rat colitis and DSS/DNBS-induced mouse models confirmed its therapeutic potential. DSM 33715 markedly attenuated body weight loss, improved macroscopic and histological scores, reduced colonic IL-1β levels, and restored goblet cell counts, paralleling the efficacy of the positive control 5-ASA [29]. It modulated gut-microbiota function by increasing beneficial genera like *Akkermansia* and *Dubosiella*, and restored SCFAs production, including acetate, butyrate, and propionate, which likely contributed to its immunomodulatory effects via GPCR signaling. As a keystone species, DSM 33715 stabilizes the microbial ecosystem, highlighting its promise as a live biotherapeutic to mitigate inflammation and promote mucosal healing in IBD [29].

*C. minuta* LPS exhibited significantly attenuated immunostimulatory effects compared to pathogenic *E. coli* LPS, functioning as a weak agonist that triggers only a mild inflammatory response in RAW 264.7 macrophages [30]. Structurally, its LPS displayed an atypical banding pattern with reduced O-antigen content, correlating with genomic deficiencies in key biosynthesis genes (e.g., missing *lpxC*, *lpxB*, and *wzy*), which underlied its low toxicity [30]. *C. minuta* LPS weakly activated the NF-κB pathway, inducing only a modest phosphorylation of IκB and p65 subunits, and subsequently elicited a substantially lower expression of pro-inflammatory cytokines, TNF-α, IL-6, and IL-1β, reduced nitric oxide (NO) and reactive oxygen species (ROS) production, and diminished macrophage proliferation and phagocytosis, all requiring concentrations 10–100 times higher than those of *E. coli* LPS to achieve measurable effects [30]. This attenuated immunogenicity positioned *C. minuta* not as a potent pathogen but as a benign commensal with potential safe applications, possibly even as an immunomodulatory agent or adjuvant, due to its inherently low endotoxic profile.

Collectively, *C. minuta* ameliorates inflammation through direct immune suppression via NF-κB/cytokine pathways, epithelial barrier restoration, and microbiome-metabolite remodeling, underscoring its potential as a live biotherapeutic for colon inflammation.

**Table 2:** Effects of *C. minuta* on microbiota, immunity, and inflammation.

Strain	Model	Dose	Duration	Change in Microbiota	Outcome	Ref.
DSM 22607	DNBS-induced colitis mice	1.5 × 10^8^ CFU/day	2 weeks	Not detected	↓ microscopic and macroscopic scores, neutrophil infiltration, activity of myeloperoxidase of colon tissue	[28]
DSM 22607	DNBS-induced colitis Sprague Dawley rat	1.5 × 10^8^ CFU/day	2 weeks	Not detected	↓ colon weight, macroscopic scores, IL-1β production	[28]
DSM 22607	DNBS-induced colitis mice	1.5 × 10^8^ CFU/day	2 weeks	↑ *Akkermansia*, *Dubosiella*, and modulated microbial metabolites in the cecum	↑ epithelial barrier ↓ inflammation, *Il-33* and *Tnfrsf8* gene expression	[52]
DSM 22607	DSS-induced colitis mice	10^9^ CFU/day	2 weeks	↑ *Lachnospiraceae_NK4A136_group*, *Dubosiella*, and ↓ *Ileibacterium*, *Parasutterella*	↓ weight loss, DAI, *Il-6* and *Tnf-α* expression, M1 macrophages ↑ IECs, *Il10* expression, M2 macrophages	[53]
DSM 33715	DNBS-induced colitis Sprague Dawley rat	10^9^ CFU/day	2 weeks	Not involved	↓ colon microscopic and macroscopic scores, IL-1β production	[29]
DSM 33715	DNBS-induced colitis mice	10^9^ CFU/day	2 weeks	Not involved	↓ colon microscopic and macroscopic scores, IL-1β production ↑ goblet cell count	[29]
DSM 33715	DSS-induced colitis mice	10^9^ CFU/day	2 weeks	Not involved	↓ body weight loss, DAI, global histological scores	[29]
DSM 22607	TNF-α-induced HT-29 cells	Culture supernatant or bacteria at MOIs of 10–50	6 h	Not involved	↓ IL-8 level, NF-κB signaling pathway	[28]
DSM 22607	TNF-α-induced Caco-2 cells	bacteria at MOI of 40	3 h	Not involved	↑ transepithelial electrical resistance	[28]
DSM 22607	Coculture of epithelial NCM460 and THP-1-derived macrophages	bacteria at MOI of 50	24 h	Not involved	↑ CD206 expression in macrophages, IL10, polarization of macrophages towards M2 phenotype	[53]
DSM 22607	RAW 264.7 macrophages	0.01–100 μg/mL *C. minuta* LPS	24 h	Not involved	*C. minuta* LPS resulted in lower levels of cellular proliferation, phagocytosis, pro-inflammatory cytokines expression, nitric oxide, reactive oxygen species production, and NF-κB activation than *E. coli* LPS	[30]

MOI: multiplicities of infection; DAI: disease activity index; ↑, increased/enriched; ↓, decreased/reduced.

### 3.3. Liver Protection

*Christensenella minuta* DSM 22607demonstrated significant efficacy in alleviating acetaminophen (APAP)-induced liver injury (AILI) through the gut–liver axis [54]. Its protective effects were evidenced by reduced serum ALT/AST levels, diminished liver necrosis, and decreased hepatocyte apoptosis in mouse models. *C. minuta* ameliorated oxidative stress, restored glutathione (GSH) levels and the GSH/GSSG ratio, enhanced superoxide dismutase (SOD) activity, and reduced lipid peroxidation [54]. It also attenuated the inflammatory response by downregulating pro-inflammatory cytokines, including IL-1β, IL-6, and TNF-α. A pivotal mechanism pathway was its regulation of phenylalanine metabolism. Elevated phenylalanine was identified as a key risk factor exacerbating AILI, associated with gut dysbiosis and increased oxidative stress [54]. *C. minuta* supplementation reversed these effects, modulating the disturbed gut microbiota by reducing the overgrowth of *Lactobacillus murinus* and restoring microbial diversity. Transcriptomic analysis revealed that hepatoprotection of *C. minuta* involved the suppression of the MAPK signaling pathway, which was activated by APAP and phenylalanine overload. *C. minuta* effectively mitigated liver damage, positioning it as a promising probiotic therapeutic for AILI [54].

*C*. *minuta* TS269500 (=DSM 22607) demonstrated significant hepatoprotective effects in a CCl_4_-induced murine liver fibrosis model, primarily by modulating gut microbiota and BA metabolism [46]. Its administration markedly alleviated hepatic injury and attenuated fibrosis by decreasing collagen deposition and downregulating expression of fibrotic markers such as α-SMA and Collagen I [46]. Additionally, *C. minuta* enrichment reshaped the gut microbiota, increasing microbial richness and specifically boosting its own abundance [46]. Mechanistically, *C. minuta* exerted its effects through high BSH activity, enhancing BA deconjugation, which altered BA profiles by reducing total hepatic BAs, increasing the ratios of secondary to primary BAs and unconjugated to conjugated BAs, and promoting fecal excretion of toxic BAs like DCA and LCA [46]. Furthermore, *C. minuta* activated the intestinal FXR/fibroblast growth factor 15 (FGF15) signaling axis, leading to suppressed hepatic expression of CYP7A1, the rate-limiting enzyme in BA synthesis, and upregulation of hepatobiliary transporters, thereby reducing BA synthesis and enhancing efflux. The indispensability of intestinal FXR signaling was confirmed by the complete abolition of *C. minuta*’s anti-fibrotic effects upon co-treatment with the FXR antagonist Gly-MCA [46]. *C. minuta* served as a pivotal microbial mediator that ameliorated liver fibrosis by regulating BA homeostasis via the gut–liver axis.

### 3.4. Gut–Brain and Gut–Ovary Axis Modulations

*Christensenella minuta* DSM 32891 demonstrated significant protective effects against chronic social defeat (CSD) stress-induced comorbidities in adolescent mice [55,55]. Administrating *C. minuta* alleviated depressive-like, anxiogenic, and antisocial behaviors, as evidenced by improved performance in behavioral tests such as the forced swimming, tail suspension, sucrose preference, and social interaction tests [55]. Additionally, it reduced cardiovascular damage and liver fibrosis associated with chronic stress. *C. minuta* modulated the hypothalamic-pituitary-adrenal (HPA) axis by enhancing acute stress responsiveness, increased corticosterone (CORT) on day 1 of CSD, and buffering chronic HPA overactivation, reduced CORT by day 10. It also prevented adrenal gland atrophy and normalized expression of glucocorticoid (NR3C1) and mineralocorticoid (NR3C2) receptors in key tissues like the striatum and colon [55]. Furthermore, *C. minuta* regulated the dopaminergic system by reducing chronic stress-induced plasma dopamine (DA) elevation, restoring DA receptor D1R and D2L expression in brain regions, and modulating DA catabolism in the gut [55]. It also exhibited anti-inflammatory effects by lowering plasma levels of CCL2 and TNF-α, reducing splenic M1 macrophages, and improving gut–barrier integrity via upregulation of tight junction proteins. Moreover, it attenuated oxidative stress in cardiac tissue by enhancing antioxidant enzyme expression and reducing DNA damage [55]. *C. minuta* induced beneficial shifts in gut-microbiota composition, increasing the abundance of taxa, such as *Desulfovibrio* and *Mucispirillum schaedleri*, which were linked to stress resilience. In summary, *C. minuta* mitigated chronic stress-induced physical and mental comorbidities through HPA axis regulation, dopaminergic pathway modulation, anti-inflammatory actions, oxidative stress reduction, and microbiota remodeling, highlighting its potential as a microbiota-based therapeutic for stress-related disorders [55].

*C. minuta* demonstrated a significant beneficial role in alleviating polycystic ovary syndrome (PCOS) through a gut-microbiota–ovary axis mechanism, primarily mediated by its metabolite butyrate [45]. Supplementation with *C. minuta* in a DHEA-induced PCOS mouse model effectively restored normal estrous cycles, improved ovarian morphology, and enhanced fertility outcomes. It also ameliorated key hormonal imbalances by significantly lowering elevated serum levels of testosterone, LH, and AMH [45]. *C. minuta* treatment improved metabolic aspects of PCOS, alleviating insulin resistance, as evidenced by improved glucose tolerance tests, insulin tolerance tests, and a reduced HOMA-IR index. *C. minuta* was a key butyrate producer, and its administration led to increased levels of butyric acid in both the feces and serum of PCOS mice [45]. Butyrate inhibited ferroptosis in ovarian granulosa cells. A transcriptomic analysis of granulosa cells from PCOS patients revealed a downregulation of the ferroptosis pathway and key antioxidant genes, such as GPX4, and SLC7A11. This was confirmed by observed elevated ferrous iron and ROS levels, alongside decreased GSH levels in these cells [45]. Butyrate supplementation counteracted this by activating the SLC7A11/GPX4 axis. Experiments on human granulosa KGN cells showed that butyrate upregulated the expression of SLC7A11 and TXNRD1, facilitating cystine uptake and ultimately restoring GPX4 activity in vitro [45]. GPX4 is crucial for reducing lipid peroxides, thereby rescuing cells from ferroptosis. This mechanism improved ovarian function and reduced the systemic symptoms of PCOS. In summary, *C. minuta* ameliorated PCOS by enriching beneficial gut microbiota, increasing production of butyrate, which in turn inhibited granulosa cell ferroptosis via the SLC7A11/TXNRD1/GPX4 pathway, leading to improved ovarian function and metabolic health [45].

## 4. Risks and Challenges of *C. minuta* Application and Future Perspective

The advent of NGPs, including strains like *C. minuta*, *Faecalibacterium prausnitzii*, and *Akkermansia muciniphila*, has expanded the therapeutic landscape by targeting more specific microbial functions and host pathways [2]. Nonetheless, the enthusiasm for novel probiotic strains is tempered by challenges in assessing their safety and efficacy. Unlike traditional probiotics, such as *Lactobacillus* and *Bifidobacterium*, which have a longstanding history of safe use, emerging strains often lack comprehensive toxicological data, standardized dosing regimens, and well-characterized mechanisms of action. Moreover, the complex interplay between introduced probiotics and the indigenous gut microbiota raises concerns about unintended microbial shifts, metabolic disturbances, or immune reactions. These uncertainties necessitate rigorous preclinical and clinical evaluations to ensure that the therapeutic benefits outweigh potential risks. There are long-term safety concerns for *C*. *minuta* in probiotic use, especially for high-risk groups, due to insufficient data on chronic exposure effects, potential dysbiosis, and immune interactions. The comprehensive understanding the biological functions of *C. minuta* and its safety profile remains limited, especially regarding its integration into complex gut ecosystems and host immune responses.

*C. minuta* is also associated with some unhealthy conditions. Compared to healthy volunteers, gut microbiota in patients with Parkinson’s disease contained higher levels of *Christensenella* and *C. minuta*, which could be associated with triggered local inflammation followed by the aggregation of α-synuclein and generation of Lewy bodies [56]. The BILIHEALTH case-control study revealed a minor and non-robust association between Gilbert’s Syndrome (GS) and *C. minuta*, and it suggests an indirect relationship potentially mediated via the lower body mass index (BMI) characteristic of GS individuals [57]. *C. minuta* has been isolated from human clinical specimens, including a mixed-infection case in the blood of a patient with acute appendicitis, co-isolated with *Desulfovibrio desulfuricans* [58]. In addition, *C. minuta* LPS can activate RAW 264.7 macrophages, inducing low-level production of pro-inflammatory cytokines, such as IL-6 and TNF-α, and reactive oxygen species via the NF-κB pathway. Though its toxicity is significantly lower than *E. coli* LPS, this activity may exacerbate inflammation in susceptible hosts [30]. These indicate its potential as an opportunistic pathogen, particularly in immunocompromised individuals or when intestinal barrier integrity is compromised.

The application of *C. minuta* as a probiotic presents risks and challenges also due to the incompletely characterized mechanisms underlying its beneficial effects, though some recent publications describe the main key mechanisms involved in the probiotic effects of *C. minuta*, including modulation on gut microbiota, modifications of BAs, the regulation of phenylalanine metabolism, and the generation of SCFAs [51,54]. For example, while *C. minuta* enhances certain bacterial groups like Lactobacillaceae, interacts with *Faecalibacterium prausnitzii*, and inhibits pathogens, the precise molecular mechanisms of cross-feeding, nutrient competition, and metabolic supplementation remain unclear, complicating dose standardization and safety assessments [23]. Additionally, its role in regulating phenylalanine metabolism and oxidative stress in conditions like acetaminophen-induced liver injury suggests complex interactions that require further investigation to avoid exacerbating dysbiosis or metabolic disruptions [54]. The scarce data of preclinical studies constitutes a hurdle for the translation of wide utilization. Long-term safety data in humans is absent, especially for high-risk groups.

The application of *C. minuta* as a probiotic faces significant risks and challenges due to its strict anaerobiosis, which complicates production, storage, and scalability. As an obligate anaerobic bacterium, *C. minuta* is highly sensitive to oxygen exposure, leading to rapid viability loss during cultivation and processing and thereby requiring specialized anaerobic equipment like gloveboxes or controlled atmospheres to maintain viability, as demonstrated in studies where flow cytometry under anaerobic conditions was essential for its isolation and culture [24]. Though it exhibits oxygen tolerance in some strains [17,28], oxygen could affect its cultivation in large-scale and biological functions [59]. This strict anaerobiosis imposes high costs and technical barriers for large-scale manufacturing, as standard probiotic production methods are often aerobic and unsuitable, potentially resulting in inconsistent product quality or reduced efficacy [25]. Additionally, the challenging culturing requirements, including the need for complex media and prolonged incubation, further hinder efficient production and commercial viability, emphasizing the necessity for advanced biotechnological solutions to overcome these obstacles for reliable probiotic formulations, as well as nanotechnology to encapsule prebiotics for survival and controlled release [24,60].

In addition to the above risks and challenges, what should also be highlighted are strain-level and strain-specific safety and function claims. Precise strain-level identification is the cornerstone of probiotic science and industrial development, directly impacting product safety, efficacy, and stability [61]. Whole-genome sequencing (WGS) acts as the gold standard for strain identification, characterizing the species and unique strain designation, and it provides a genetic background for comprehensive strain-level safety assessments. *C. minuta* exhibits strain-specific effects [29]. DSM 33407 and DSM 22607 are the most reported strains, and more data is needed to elucidate the distinction and applicability of different strains. There is an urgent need for clinical translation research, even large-scale human clinical trials, to validate *C. minuta*’s efficacy in metabolic diseases and IBD, focusing on optimal dosing, long-term safety, and high-risk populations. Xla1, a *C. minuta*-based single-strain LBP, is recorded for Phase I human trial to evaluate safety, tolerability, and the impact on gut microbiota in healthy, overweight, and obese adults [59,62], and the results are not yet available.

## 5. Conclusions

*Christensenella minuta* demonstrates substantial potential as a next-generation probiotic, with evidenced capabilities in modulating host metabolism, immunity, and gut-barrier integrity. Its beneficial effects are mediated through multiple mechanisms, including the production of unique BA derivatives, SCFAs, and competitive microbial interactions.

The bacterium’s therapeutic promise is evident across a range of conditions, such as metabolic disorders, inflammatory bowel disease, and liver injury, highlighting its systemic influence. Nevertheless, several challenges must be addressed to enable clinical translation. These include its oxygen-sensitive nature complicating production and storage, strain-specific functional variations requiring precise characterization, and insufficient long-term safety data, particularly concerning vulnerable populations.

Future efforts should focus on advancing bioprocessing technologies to ensure viability, conducting well-designed human trials to confirm efficacy and safety, and deepening the mechanistic understanding of its interactions with resident microbiota. Through targeted research and development, *C. minuta* can evolve from a scientific prospect into an effective biotherapeutic agent for addressing various chronic diseases.

## Data Availability

No new data were created or analyzed in this study. Data sharing is not applicable to this article.

## Abbreviations

The following abbreviations are used in this manuscript:

NGPs	Next-generation probiotics
LPS	Lipopolysaccharide
BA	Bile acid
FXR	Farnesoid X receptor
SCFAs	Short-chain fatty acids
